# On the Variability of the Barometric Effect and Its Relation to Cosmic-Ray Neutron Sensing

**DOI:** 10.3390/s26030925

**Published:** 2026-02-01

**Authors:** Patrick Davies, Roland Baatz, Paul Schattan, Emmanuel Quansah, Leonard Kofitse Amekudzi, Heye Reemt Bogena

**Affiliations:** 1 Department of Meteorology and Climate Science, Kwame Nkrumah University of Science and Technology, Kumasi AK-385-1973, Ghana; 2 CDM Smith SE, Bouchestr. 12, 12435 Berlin, Germany; 3 Leibniz Centre for Agricultural Landscape Research (ZALF), Eberswalder Str. 84, 15374 Muncheberg, Germany; 4 Institute of Geography, University of Innsbruck, Innrain 52f, 6020 Innsbruck, Austria; 5 Institute of Hydrology and Water Management (HyWa), Universität für Bodenkultur Wien, Muthgasse 18, 1190 Vienna, Austria; 6 Institute of Bio- and Geosciences, Agrosphere (IBG-3), Forschungszentrum Jülich GmbH, 52428 Jülich, Germany

**Keywords:** barometric coefficient, cosmic-ray neutron sensing, cut-off rigidity

## Abstract

Accurate estimation of the barometric coefficient (β) is important for correcting pressure effects in soil moisture data from cosmic-ray neutron sensing (CRNS) due to the barometric effect. To evaluate estimation strategies for β, we compared analytical and empirical approaches using 71 CRNS and 46 neutron monitor (NM) stations across the United States, Europe, and globally. Our results show spatio-temporal variation in the barometric effect, with β ranging from 0.66 to 0.82 %hPa for NM and from 0.63 to 0.80 %hPa for CRNS. These coefficients exhibit higher variability than previously published semi-analytical models. In addition, we found that the analytically determined β values were systematically lower compared with empirical estimates, with stronger agreement between the two empirical methods (r≈0.67) than between empirical and analytical approaches. Furthermore, NM stations produced higher β values than CRNS, indicating that differences in detector energy sensitivity affected the values of β. Principal Component Analysis (PCA) further showed that the analytical and empirical β estimates clustered together, reflecting shared sensitivity to elevation. In contrast, soil moisture and atmospheric humidity projected nearly orthogonally to the β vectors, indicating negligible influence, while cut-off rigidity contributed to a separate, inverse gradient. Analytical β estimates were fully orthogonal to AH, while empirical methods showed only slight deviations beyond orthogonality. The barometric coefficient (β), therefore, varies with location, altitude, atmospheric conditions, and sensor type, highlighting the necessity of station-specific values for precise correction. Overall, our study emphasizes the need for atmospheric correction in CRNS measurements and introduces a method for deriving site- and sensor-specific β values for accurate soil moisture estimation.

## 1. Introduction

The cosmic-ray neutron sensing (CRNS) method [1,2] effectively estimates soil water content (SWC) based on the inverse relationship between cosmic-ray neutron intensity near the land surface and hydrogen present in soil water. The method is commonly used at the field scale [3] and can also be useful at the continental scale by integrating CRNS networks [4].

Cosmic-ray neutrons are secondary particles produced by inelastic collisions of high-energy primary particles from outer space with Earth’s atmospheric nuclei [5] which are then moderated by elastic collisions with hydrogen nuclei [6]. Inelastic collision processes lead to the attenuation of the nucleon cascade, and their effectiveness depends on the mass thickness of the atmosphere, which is typically represented by the barometric pressure. Since the barometric pressure of the atmosphere fluctuates over time with changing weather conditions, substantial temporal fluctuations occur in the neutron count rate of a CRNS detector. Consequently, the correction for the barometric effect is one of the most important data corrections for CRNS [2], for which reason CRNS sensors are always equipped with a pressure sensor [7]. In addition, fluctuations in the solar and geomagnetic fields modulate the amount of high-energy cosmic rays penetrating the atmosphere, which leads to further changes in the intensity of cosmic-ray neutrons [8,9]. Temporal variations in cosmic-ray neutrons due to these influences are detected with neutron monitors (NMs) [10], which are sensitive to high-energy secondary neutrons but insensitive to local environmental factors such as SWC. Therefore, NM data are typically used to correct CRNS raw data from fluctuations in incoming neutrons [11].

Although the general dependence of barometric coefficients on geomagnetic latitude and altitude has been known for some time [12], in CRNS applications, as an approximation, the coefficient is often assumed to be constant in time and space. For example, a β coefficient of 0.76 % hPa suggested by [13] was generally applied across the COSMOS-Europe network [4]. Notably, the range of β estimates for COSMOS-Europe sites in a recent data-driven study [14] was considerably broader, spanning 0.52 to 0.78 %hPa (with a mean of 0.71 %hPa and a median of 0.73 %hPa), than the commonly utilized reference value of 0.76 %hPa. Studies have shown that the barometric coefficient is a complex variable that depends on factors such as geographical latitude, altitude, type and energy of incoming primary particles [14,15,16], and solar activity [6,17]. In addition, the barometric coefficient depends on the energy sensitivity of the detector [18], as the nucleon energy spectrum shifts towards lower energies as the barometric coefficients decrease [19]. Therefore, since the energy range covered by NMs (∼0.5 to 100 GV; [18]) differs strongly from CRNS (∼0.1 eV to 1 MeV; [20]), it can be assumed that the β determined with neutron count rates observed by CRNS detectors and NMs are also different. In the past, analytical relationships of β have been mainly derived from selected neutron monitor locations across a wide range of geomagnetic cut-off rigidities but with varying altitudes [13,16,21,22,23]. Further relations were established using data from ship-based latitude surveys [24,25]. In addition to solely minimizing the correlation between incident radiation and atmospheric pressure [26], some studies also included the normalization of the data with a reference NM to remove effects of variations in the incoming comsic-ray flux [16,27]. A study incorporating different neutron detection energies [19] found that the attenuation length (i.e., the inverse of β) ranged from 128 to 142 gcm^−2^ at altitudes between sea level and 5000 m when using NM data, whereas attenuation lengths derived from thermal neutron data are somewhat higher, ranging from 134 to 155 gcm^−2^ at similar altitudes. For Europe, the difference in β is small when using a single analytical function [4]. Still, the results for a location at a cut-off rigidity (CR) of 4.5 GV would vary from an attenuation length of 132 to 142 gcm^−2^ when using different analytical equations [16,24,25].

Therefore, this study proposes a site- and energy-specific estimation of β using local CRNS data instead of a global fit based on NM data to obtain more suitable values for β in the effective energy range of CRNS sensors. Additionally, we compare the values of β obtained using our new method with those determined by three empirical and analytical methods. This study is the first to jointly determine β coefficients for numerous CRNS (71 stations in Europe and the USA) and NM (46 globally) stations and analyze their spatio-temporal variability.

## 2. Materials and Methods

Our methodological framework is designed to determine β coefficients from hourly, long-term records of cosmic-ray intensity measured with neutron detectors. The approach combines two analytical methods and two empirical methods (labeled A and B), allowing for both theoretical derivation and data-driven estimation. For the empirical component, we draw upon extensive observational datasets:

- Cosmic-ray neutron sensing (CRNS) networks comprising 71 stations across the United States [2] and Europe [4].
- Neutron Monitor Database (NMDB), which provides data from 46 stations worldwide (https://www.nmdb.eu/).

The NMDB archive spans 1958–2023 and covers sites with geomagnetic cut-off rigidities between 0 and 16.8 GeV, while the CRNS stations fall within the narrower range of 1–7.5 GeV. Together, these datasets provide a robust basis for performing evaluation across diverse spatial and temporal conditions.

### 2.1. Data Processing and Quality Control

The data-driven empirical methods (A and B) were applied following standard CRNS corrections (humidity and incoming neutron intensity) and the filtering of neutron count data from CRNS and NM stations to address known environmental influences and to ensure data comparability across sites. The quality criteria included removing data with unrealistic temporal behavior due to sensor malfunctioning, as well as values out of the physical ranges for air humidity, air pressure, and neutron flux. Additionally, months with fewer than 18 days of daily neutron observations were considered insufficiently representative and were excluded from the analysis. The filtered data were then corrected for fluctuations in the incoming cosmic-ray neutron intensity, which can introduce temporal noise unrelated to local atmospheric conditions. We applied the correction approach developed by [11], ensuring that both the NM and CRNS datasets reflected consistent background intensity levels.

**Figure 1:**
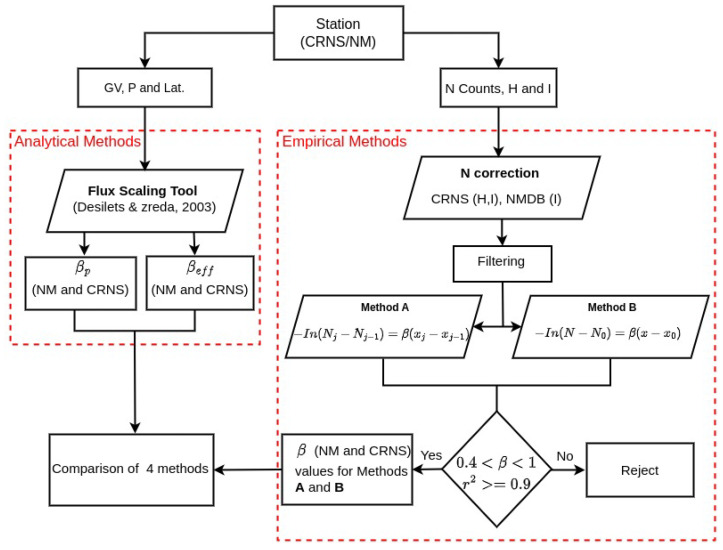
Flowchart of CRNS and NMDB data processing for β calculation.

For CRNS stations, an additional correction was applied to account for atmospheric humidity [28], which attenuates neutron flux and can bias measurements if left unadjusted. This humidity correction was implemented using site-specific humidity records and established calibration procedures. Following these corrections, the relationship (barometric coefficient) between neutron counts and atmospheric pressure was estimated for each station using the two empirical methods (A and B) and the analytical approach by [19]. Figure 1 summarizes the overall workflow, including input variables, correction steps, and the branching into analytical scaling and empirical regression approaches to estimating β.

### 2.2. Methods

#### Data-Driven Approaches to Barometric Coefficient Estimation

The influence of atmospheric pressure on the flux of cosmic-ray neutrons *N* (i.e., the barometric effect) can be corrected using the following Equation (1) described in [29,30].

(1) $$N_{p}=N\times e^{\beta (P-P_{ref})}$$

where $P_{ref}$ is the reference atmospheric pressure, *P* is the atmospheric pressure at the time of observation in *hPa*, $N_{p}$ is the corrected neutron count rate, and β is the barometric coefficient in %hPa. By integrating Equation (1), we obtain an Equation (2), which allow us to infer the β coefficient as the slope of a linear regression:

(2) $$-ln\left(N_{p}-N\right)=\beta \left(P-P_{ref}\right)$$

The derived equation was applied in previous studies to the NM dataset to estimate the β coefficient [27].

Building on the pressure-based formulation in Equation (2), we developed two empirical methods for estimating β directly from corrected neutron count data. This equation expresses the logarithmic relationship between the pressure-adjusted neutron flux and deviations from a reference pressure, providing a physically grounded basis for regression-based estimation. By adapting this structure to station-level time series, we formulated two empirical variants (Methods A and B) that differ in how they define reference states and temporal increments. The atmospheric air pressure (*P*) was converted into atmospheric depth *x* (gcm^−2^) based on gravity (*g*) using Equation (3) as described in [11] for the two empirical approaches.

(3) x=10×P/g

Method A is based on Equation (2) to estimate daily β from corrected neutron count rates, as shown in Equation (4):

(4) $$-ln\left(N_{hi(j)}-N_{hi(j-1)}\right)=\beta \left(x_{\left(j\right)}-x_{\left(j-1\right)}\right)$$

where $N_{hi(j)}$ and $N_{hi(j-1)}$ are the corrected neutron count rates and atmospheric depths *x* in gcm^−2^ on day *j* and on the previous day, j−1, respectively. To minimize the impact of changes in ambient water, neutron counts that deviated by more than 10% from the previous and subsequent readings were excluded. Method A is consequently less susceptible to changes in soil moisture and is therefore particularly suitable for ground-based CRNS data. Empirical method B adopts the approach proposed by [23,27] to estimate β, as shown in Equation (5):

(5) $$-ln\left(N_{p}-N_{ref}\right)=\beta \left(x-x_{ref}\right)$$

where $N_{ref}$ and $x_{ref}$ are the average observations of hourly neutron counts and atmospheric depth. Note that for both empirical methods, monthly β were estimated using CRNS and NM data, as suggested by [23]. To ensure high accuracy of the estimated β values, only regressions with $r^{2}>=0.9$ and β between 0.004 and 0.02 %hPa are considered valid (see Figure A3 and Figure A4 for method A’s regression). The resulting monthly β estimates are then averaged to obtain a mean β and standard deviation (σ) for the respective CRNS and NM stations. This approach, based on a large number of individual monthly values, results in more robust β estimates, reducing the statistical noise expected, e.g., from changes in ambient moisture conditions.

### 2.3. Analytical Methods

The analytical method developed by [19] has become the predominant approach for determining β in the past decade. This approach, based on previous studies, e.g., [12,31], determines the barometric coefficient at a certain atmospheric depth ($\beta_{p}$) by utilizing the theoretical relation between the coefficient and its key affecting elements, namely, cut-off rigidity and altitude ([12]):

(6) $$\beta_{A}=n{\left(1+e^{\left(-\alpha CR^{k}\right)}\right)}^{-1}x+\left(b_{0}+b_{1}CR+b_{2}CR^{2}\right)x^{2}+\left(b_{3}+b_{4}CR+b_{5}CR^{2}\right)x^{3}$$

To further estimate the effective barometric coefficient ($\beta_{A,eff}$) between two atmospheric depths $x_{1}$ and $x_{2}$, Equation (7) is used as follows:

(7) $$\beta_{A,eff}=x_{2}-x_{1}{\left[n{\left(1+e^{\left(-\alpha CR^{k}\right)}\right)}^{-1}x+\frac{\left(b_{0}+b_{1}CR+b_{2}CR^{2}\right)x^{2}}{2}+\frac{\left(b_{3}+b_{4}CR+b_{5}CR^{2}\right)x^{3}}{3}+\frac{\left(b_{6}+b_{7}CR+b_{8}CR^{2}\right)x^{4}}{4}\right]}_{x_{1}}^{x_{2}}$$

where $\beta_{A}$ and $\beta_{A,eff}$ are dependent on the location (cut-off rigidity (CR) and latitude (Lat)) and the atmospheric depth *x* in gcm^−2^, α=0.055, k=0.601, and n=0.123. Each site’s atmospheric depth (*x*) is derived from pressure information using Equation (3). This analytical approach is also available online as a ‘Flux scaling tool’ (https://crnslab.org), which provides yearly estimates of β based on longitude, altitude, and latitude. In this study, we applied the analytical framework described in [19] using site-specific monthly pressure data rather than annual averages. Monthly pressure measurements were converted into altitude using Equation (7), and β was calculated for each month following the same theoretical framework. This approach allows us to capture temporal variability associated with seasonal pressure patterns while maintaining consistency with the established analytical method.

### 2.4. Principal Component Analysis (PCA)

Principal Component Analysis was applied to identify orthogonal modes of variability in the relationship between the CRNS β coefficient and environmental covariates. For each site i∈{1,…,N}, we obtained method-specific estimates of parameter β: analytical ($\beta_{i,(eff)}$), empirical A ($\beta_{i,(EM-A)}$), and empirical B ($\beta_{i,(EM-B)}$). In addition, each site was characterized by environmental and geophysical variables, including soil moisture ($S_{i}$), atmospheric humidity ($AH_{i}$), and geomagnetic cut-off rigidity ($CR_{i}$). These values were assembled into a data matrix:

(8) $$Z=\left[\begin{array}{cccccc} \beta_{1,\left(A,eff\right)} & \beta_{1,(EM-A)} & \beta_{1,(EM-B)} & SM_{1} & AH_{1} & CR_{1}\\ \beta_{2,\left(A,eff\right)} & \beta_{2,(EM-A)} & \beta_{2,(EM-B)} & SM_{2} & AH_{2} & CR_{2}\\ \vdots & \vdots & \vdots & \vdots & \vdots & \vdots \\ \beta_{N,\left(A,eff\right)} & \beta_{N,(EM-A)} & \beta_{N,(EM-B)} & SM_{N} & AH_{N} & CR_{N} \end{array}\right].$$

The variables were standardized to ensure comparability across scales. Principal Component Analysis was then applied to the standardized matrix to reduce dimensionality and identify dominant modes of variability. The covariance matrix was computed as

(9) $$S=\frac{1}{N-1}X^{⊤}X,$$

where *X* is the standardized data matrix. Eigendecomposition of *S* yielded orthogonal loading vectors and associated eigenvalues, which define the principal components. Site scores were obtained by projecting the standardized data onto the loading vectors. The proportion of variance explained by each component was calculated as

(10) $${\mathrm{EVR}}_{k}=\frac{\lambda_{k}}{\sum_{j=1}^{p}\lambda_{j}},$$

where $\lambda_{k}$ is the eigenvalue of component *k*. For visualization, the first two principal components were retained, and a biplot was constructed. In this representation, site scores are plotted in the PC1–PC2 space, while variable loadings are displayed as vectors scaled by the square root of their corresponding eigenvalues. This allows for the simultaneous visualization of site clustering and the relative contributions of method-specific β estimates and geophysical variables.

## 3. Results

**Figure 2:**
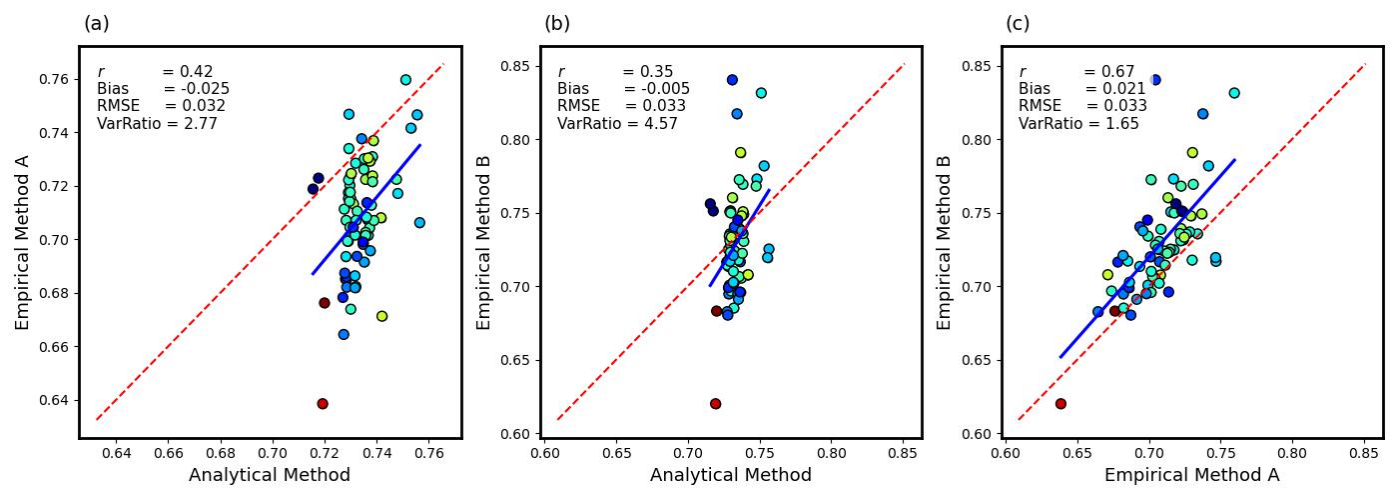
Pairwise scatter plots comparing analytical and empirical estimation methods. (**a**) Relationship between empirical method A and the analytical method. (**b**) Relationship between empirical method B and the analytical method. (**c**) Relationship between empirical method B and empirical method A. Data points are colored according to the compression ratio (CR), with values ranging from 0.1 (blue) to 6 (red).

The agreement between the analytical and empirical approaches for estimating β is shown in Figure 2, with scatter plots (Figure 2a–c) comparing the CRNS-estimated β values derived from the analytical method (the proposed method; $\beta_{A,eff}$) with those from two empirical methods (A and B), as well as the comparison between the two empirical methods. Figure 2a, the comparison between the analytical method ($\beta_{A,eff}$) and empirical method A ($\beta_{EM-A}$), reveals a moderate positive correlation (r=0.42), RMSE=0.032 and a variability ratio of 2.77, indicating some agreement with analytical β but also noticeable scatter, particularly at lower β values. A slightly weaker correlation (r = 0.35) is observed between the analytical method and empirical method B (see Figure 2b), with RMSE = 0.033, and a higher variability ratio of 4.57, reflecting greater spread and overestimation of variance despite its lower bias. In contrast, Figure 2c demonstrates a stronger relationship between the two empirical methods (r=0.67, RMSE=0.033, and variability ratio = 1.65), suggesting greater consistency between empirical approaches than between empirical and analytical methods. Overall, both empirical methods show limited agreement with the analytical reference, but method A performs slightly better by combining lower RMSE, stronger correlation, and more moderate variance. Given that $\beta_{EM-A}$ aligns more closely with the analytical method and provides more stable estimates than method B, we present results from empirical method A to provide a balanced and consistent perspective on β.

### 3.1. Estimated Barometric Coefficients Using Empirical and Analytical Methods

**Figure 3:**
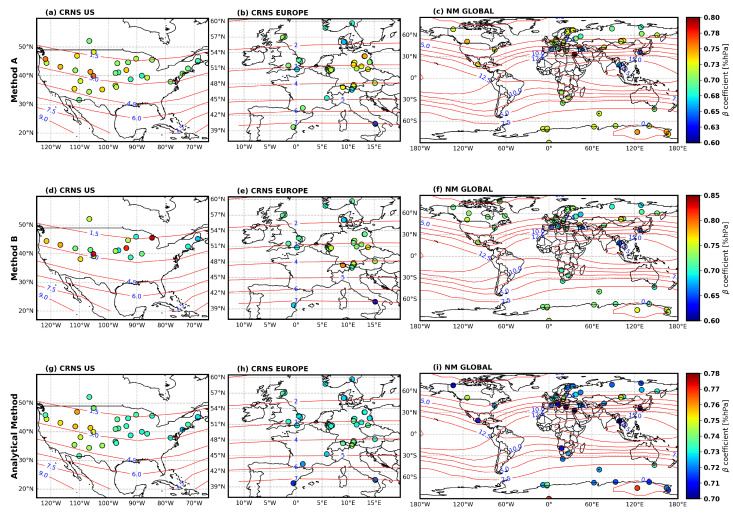
Geographical distribution of β from CRNS data in the US (column 1), CRNS data in Europe (column 2) and NM global data (column 3) estimated using empirical method A (row 1), method B (row 2), and the analytical method (row 3). The red contour lines represent geomagnetic cut-off rigidity values.

Figure 3 presents the spatial distribution of monthly averaged β coefficients derived from three methods (analytical and empirical methods A and B) over a period for CRNS stations in the US and Europe, as well as NM stations globally. The red contour lines represent geomagnetic cut-off rigidity values, providing context for the spatial variability in atmospheric pressure corrections. The results from empirical method A ($\beta_{EM-A}$) reveal the average of monthly β coefficients ranging from 0.60 to 0.80 across all networks, with the majority of CRNS stations clustering between 0.65 and 0.75. In the US, approximately 95% of stations exhibit values within this range, while European stations (≈96%) show similar β values. The global NM network demonstrates comparable ranges, though out of 47 NM stations, 4 stations recorded an average of monthly β<0.70 %hPa (see Figure 3a–c).

The average β estimates derived from CRNS data were 0.71±0.020 %hPa and 0.71±0.021 %hPa for stations in the US and Europe, respectively. Analysis of global NM data gave an average of 0.72±0.030 (Table 1). Method B ($\beta_{EM-B}$) shows a similar range but extends the upper limit to 0.85 %hPa compared with method A. US CRNS stations show the most pronounced increase for some locations, exceeding 0.75, particularly in the northern part. European stations maintain their spatial patterns but with enhanced magnitude, while the global NM distribution exhibits marginal shifts toward higher values in the Northern Hemisphere mid-latitudes. $\beta_{EM-B}$ yields comparable average values across all networks, with 0.75±0.040 %hPa for US CRNS, 0.72±0.026 %hPa for Europe CRNS, and 0.72±0.024 for the global NM network (Table 1).

The analytical method (Figure 3g–i) produced a more constrained range of averaged monthly estimated β (0.70–0.75 %hPa) compared with empirical methods A and B. Both US and Europe CRNS stations showed less variation in the averaged monthly β between 0.7 and 0.76 %hPa. European stations were clustered tightly around 0.73–0.75, and the global NM network showed a similarly reduced variability pattern. All three methods showed a pronounced longitudinal decrease from the western part of the US (120°W) to the east (70°W) (see Figure 3a,d,g), although the range of the analytical β variability is smaller than that of the empirical methods. Strong regional contrasts are observed for Europe, with higher empirical barometric coefficient ($\beta_{EM-A}$ and $\beta_{EM-B}$) values in the continental interior (0.68–0.76 %hPa at 50°N–54°N) compared with the analytical method. At the global scale, a clear indication of an inverse relation between the cut-off rigidity and the estimated β coefficient was observed using the NM (see Figure 3c,f,i). Overall, β values exhibit relatively consistent ranges across methods and regions, with only minor differences between empirical and analytical estimates. The β values for CRNS (in both the US and EU regions) and for NM sensors (at the global scale) are quite similar across all methods presented.

**Figure A1:**
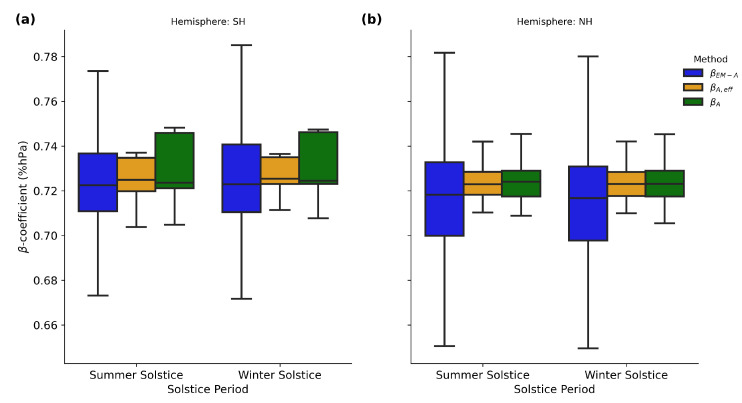
Seasonal comparison of the distribution of β coefficients during solstice periods (winter and summer) for (**a**) Southern Hemisphere (SH) and (**b**) Northern Hemisphere (NH).

Furthermore, we compared β estimates during summer and winter solstice periods across both hemispheres (Figure A1). This analysis is only possible due to the global distribution of neutron monitor stations. Both hemispheres show stable β values across seasons, with Southern Hemisphere medians at 0.72–0.75 %hPa and Northern Hemisphere medians at 0.72–0.73 %hPa. All three methods show consistent behavior, with $\beta_{A}$ producing slightly higher values. This seasonal stability indicates that barometric sensitivity does not change substantially with seasonal atmospheric variations.

### 3.2. Variability of β Between Stations and Correlation with Cut-Off Rigidity

**Figure 4:**
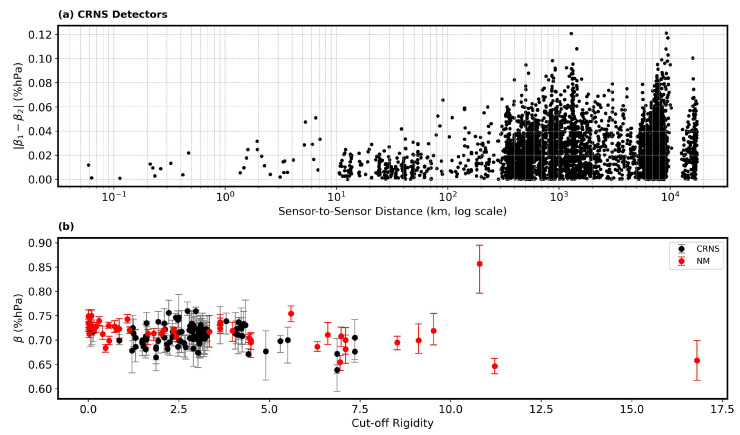
β estimates for all CRNS stations. (**a**) The relationship of β-to-β and sensor-to-sensor distance. Sensors in close range are expected to exhibit little difference, with higher variability observed at larger distances. (**b**) β for CRNS (black) and NM (red) in relation to geomagnetic cut-off rigidity.

Figure 4a shows the absolute difference in β between stations as a function of the distance between the stations on a logarithmic scale. The results show that the absolute β difference remains small when stations are close together (typically less than 0.02) and increases with the increase in distance. This suggests that barometric sensitivity remains relatively unchanged within 10 km, but the spread in β differences becomes more pronounced at greater distances, reaching values up to 0.12 %hPa. This trend suggests that spatial distance between stations contributes to increased β variability, although most differences remain relatively modest even at the largest separations.

Figure 4b shows the dependence of β on the geomagnetic cut-off rigidity for both CRNS stations (black points) and NM stations (red points). Across the entire range of cut-off rigidities, both datasets show a general clustering of β values between 0.65 and 0.80. Furthermore, there is no strong monotonic trend in β with the increase in cut-off rigidity, though a slight decrease in β is observed at higher rigidities, particularly for the NM stations. The error bars indicate the standard error for each measurement, highlighting that the observed spread is not solely due to measurement uncertainty. Generally, higher β values are observed for NM compared with CRNS detectors. Overall, these results demonstrate that while β values are broadly consistent across stations and detector type, both spatial separation and geomagnetic cut-off rigidity introduce measurable, though generally moderate, variability in β. This emphasizes the importance of considering both geographic and geomagnetic factors when comparing or aggregating β for different locations.

**Figure A2:**
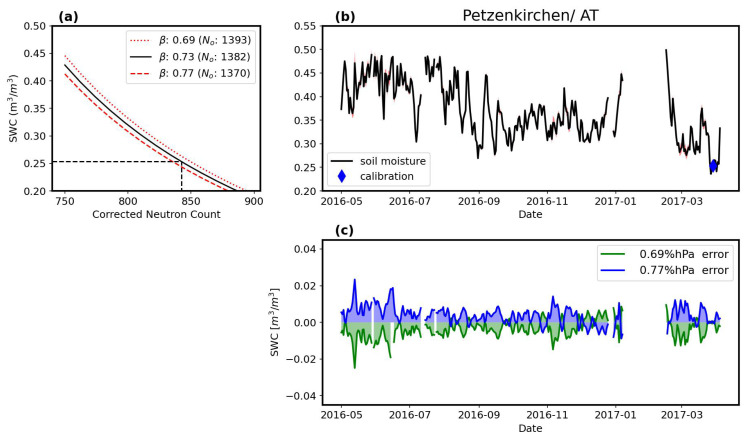
Sensitivity of different β coefficients to the $N_{0}$ calibration parameter (**a**). The generated time series of soil moisture from three β (**b**) and associated uncertainty considering a β value of 0.74 %hPa as the true β coefficient of Petzenkirchen, Austria (**c**).

**Figure A3:**
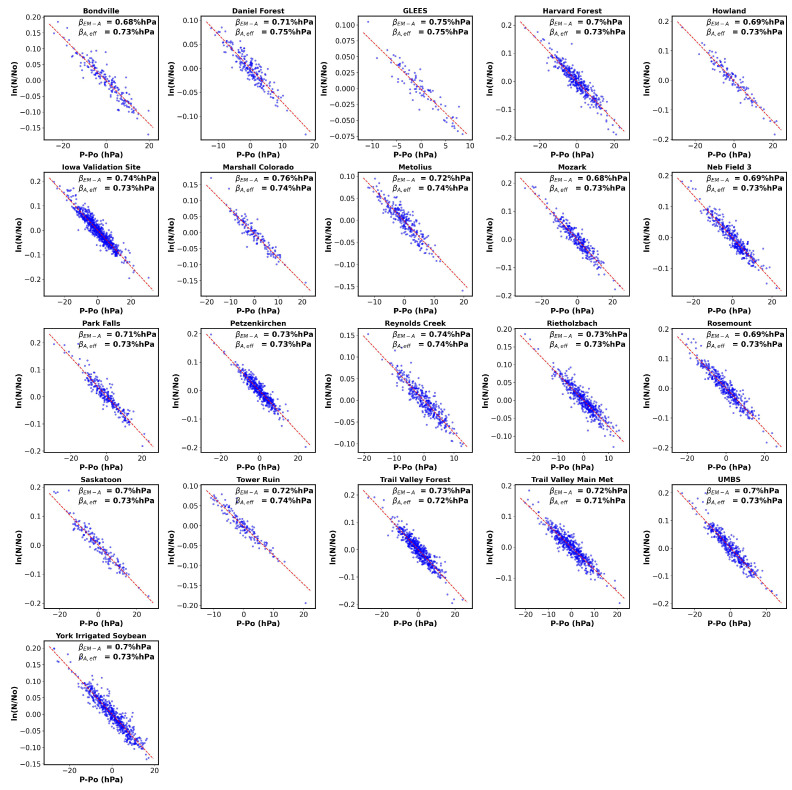
Regression between the relative neutron count rates and atmospheric pressure for COSMOS-US stations using empirical method A.

**Figure A4:**
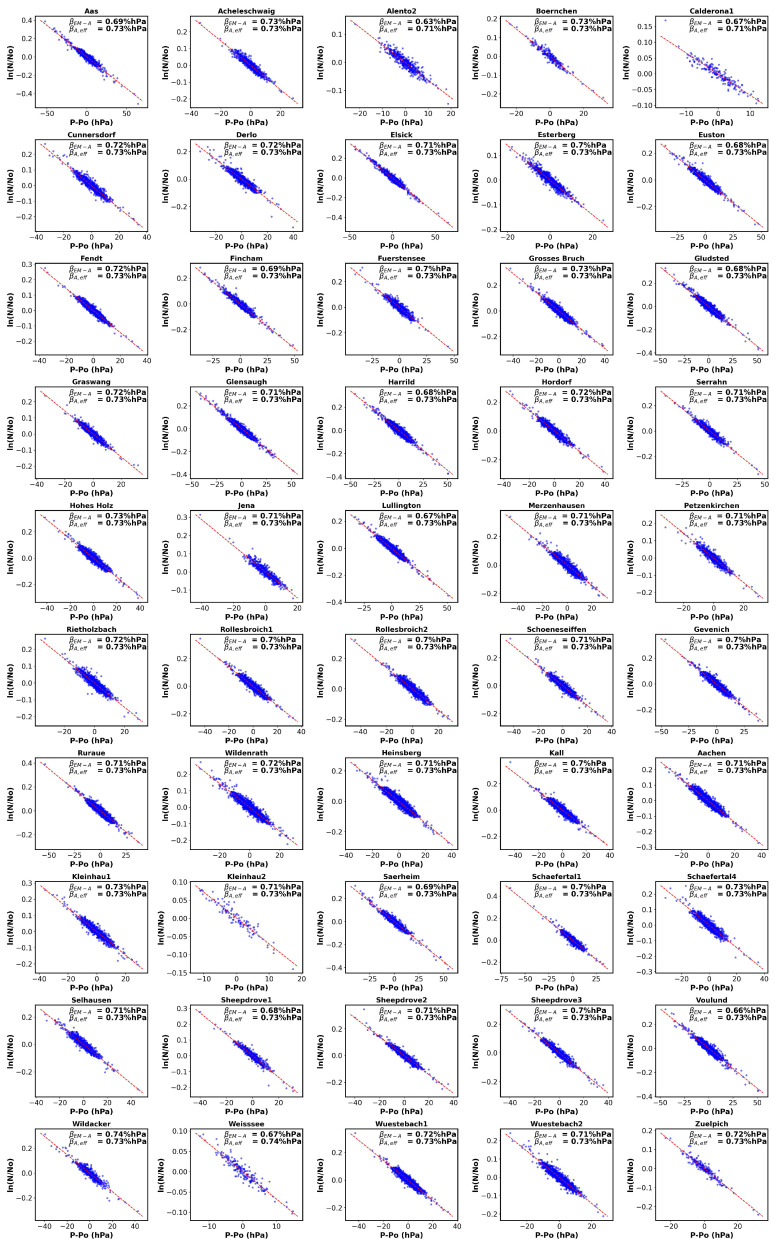
Regression between the relative neutron count rates and atmospheric pressure for the selected COSMOS-Europe stations using empirical method A.

To quantify the practical significance of this spatial variability, we conducted a sensitivity analysis at Petzenkirchen, Austria. We calibrated CRNS models using three β values spanning the sites’ monthly estimated range, 0.69 %hPa (site mean), 0.73 %hPa (upper extreme), and 0.77 %hPa (lower extreme), yielding $N_{0}$ parameters of 1393, 1382, and 1397, respectively. The resulting soil moisture estimates showed systematic biases of ±0.01–0.02 m^3^/m^3^ throughout the time series (Appendix A Figure A2). These differences were moisture-dependent, reaching ±0.02 m^3^/m^3^ during wet periods and compressing to ±0.005 m^3^/m^3^ under dry conditions. This demonstrates that a β variation of 10%, comparable to the spatial variability observed across our network, translates into soil moisture errors comparable to CRNS target accuracy. The systematic nature of these biases indicates that using inappropriate β values introduces persistent structural errors that cannot be corrected without site-specific recalibration, underscoring the importance of accurate β determination for operational CRNS networks.

### 3.3. Covariance Structure of (β) and Its Influencing Factors

**Figure 5:**
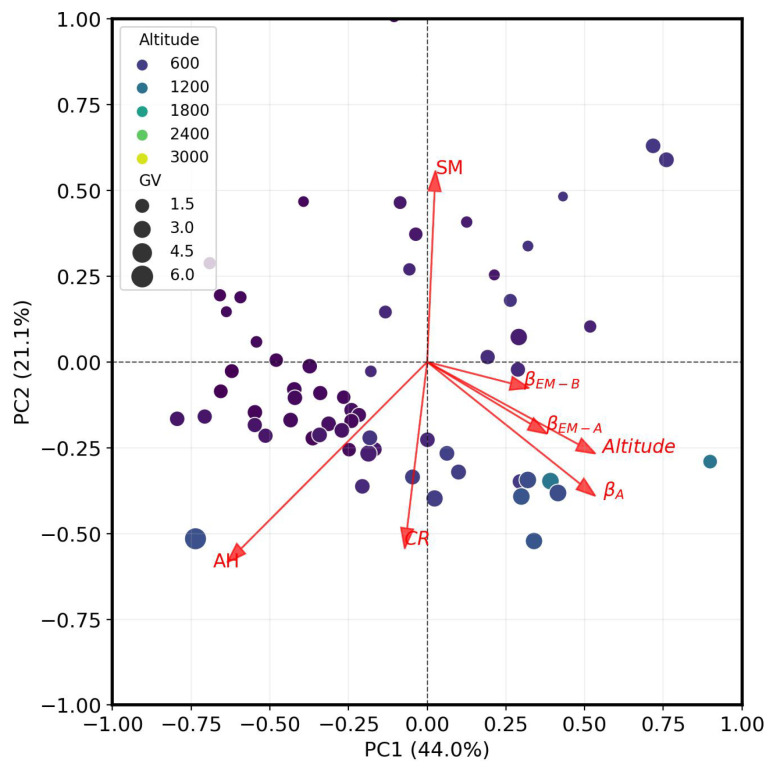
PCA biplot showing the association between site variables (e.g., altitude, CR, SM, and AH) and the three β estimation methods ($\beta_{A}$, $\beta_{EM-A}$, and $\beta_{EM-B}$). The colors of the points represent increasing altitude, it size is represented by increasing cut-off rigidity (CR).

Figure 5 presents the PCA biplot illustrating the relationships among barometric coefficient estimates ($\beta_{A}$, $\beta_{EM-A}$, and $\beta_{EM-B}$) and key environmental variables (altitude, cut-off rigidity (CR), absolute humidity (AH), and soil moisture (SM)). The first two principal components explain 65.1% of the total variance (44.0% by PC1 and 21.1% by PC2). The PCA biplot reveals the distinct geographical structuring of the dataset. The vector represents altitude projects strongly along the positive PC1 axis. The CR, in contrast, points almost due south in the third quadrant (negative PC1 and negative PC2), suggesting that it contributes to a separate, inverse geographical gradient. SM projects nearly vertically along the positive PC2 axis, while AH points into the lower-left quadrant, forming an approximate angle of 225° and thus contributes to variance orthogonal to both altitude and SM. The spatial distribution of site scores reflects these patterns, with samples stratified along PC1 according to altitude and dispersed along PC2 in relation to SM.

The three β estimates occupy the same quadrant of the PCA space-positive PC1 and negative PC2 but exhibit nuanced directional differences. $\beta_{\mathrm{EM}-A}$ is positioned between $\beta_{A}$ and altitude, indicating that it shares variance with both but is slightly more aligned with the analytical method. $\beta_{\mathrm{EM}-B}$ lies near the PC2 = 0 axis, close to altitude, but is separated by an angle of approximately 15°, suggesting a modest divergence in directional loading. $\beta_{A}$ itself is strongly aligned with altitude, reinforcing its sensitivity to elevation-related factors. The proximity of $\beta_{\mathrm{EM}-A}$ and $\beta_{\mathrm{EM}-B}$ in PCA space confirms their internal consistency, while their relative positions to $\beta_{A}$ reflect method-specific contrasts. The empirical methods appear to converge toward the analytical baseline along the altitude-driven PC1 axis, but retain distinct orientations that suggest differential responsiveness to geophysical inputs. The vector lengths of all three β estimates are substantial, indicating that each method makes a meaningful contribution to the overall variance structure.

## 4. Discussion

This study assessed barometric coefficients (β) across multiple CRNS and NM stations using analytical and empirical approaches. Results show strong spatio-temporal variability in β, with NM stations consistently yielding higher β values than CRNS stations. Analytical estimates ($\beta_{A}$ and $\beta_{eff}$) are systematically higher than empirical methods (EM-A and EM-B), with notable discrepancies at lower β values. According to [32], barometric coefficients typically fall between 0.76 and 0.68 %hPa. Our empirically estimated values align well with this range, confirming their physical plausibility. Notably, previous studies have reported systematically lower coefficients, with the commonly utilized reference value being −0.76 %hPa [14,33,34]. These empirical estimates show some consistency with the established range and at least provide a step toward locally and sensor-specific derived β to improve the CRNS soil moisture estimate. Comparison among methods shows that empirical approaches are more consistent with each other than with analytical methods. However, $\beta_{\mathrm{EM}-B}$ tends to overestimate relative to both analytical and EM-A, whereas $\beta_{\mathrm{EM}-A}$ aligns more closely with analytical estimates and provides more conservative corrections. PCA further reveals altitude and humidity as the dominant controls on β variability (PC1: 44%), with cut-off rigidity (CR) and soil moisture (SM) explaining secondary variance (PC2: 21%).

The higher β values observed at NM stations relative to CRNS may be due to differences in detector energy sensitivity: NM stations detect higher-energy neutrons, while CRNS are more sensitive to moderated, lower-energy neutrons. This agrees with observations from Antarctic NM studies by [35], which similarly reported elevated β values for NM detectors. Analytical methods systematically underestimate β compared with empirical approaches because they depend solely on altitude and cut-off rigidity, neglecting local atmospheric and hydrological variability that empirical methods implicitly capture. Similar discrepancies between analytical and empirical methods have been reported by [16,27], attributed to differences in environmental sensitivity and the temporal resolution of pressure data (e.g., hourly versus daily anomalies).

The covariance contributions from the PCA provide insight into how the three β estimation methods co-vary across sites and their relationship to environmental gradients. PC1 and PC2 explain 64.1% of total variance (44.0% and 21.1%, respectively) in the combined dataset. It is essential to emphasize that PCA identifies how variables change together across sites, rather than establishing causal mechanisms. All three β estimates ($\beta_{A}$, $\beta_{EM-A}$, and $\beta_{EM-B}$) project into the same quadrant (positive PC1 and negative PC2), indicating shared spatial variance structure. $\beta_{A}$ and $\beta_{EM-A}$ nearly overlap, while $\beta_{EM-B}$ shows modest angular separation. This clustering demonstrates that the methods rank sites similarly: sites yielding high β values with one method tend to yield high values with the others. Despite different approaches (analytical foundation, calibrations, and statistical approach), the methods converge in their spatial patterns. This co-alignment is significant: substantial angular separation would indicate that empirical calibration fundamentally restructures β’s response to environmental conditions, but the observed alignment shows that the empirical methods do not fundamentally alter the spatial variance structure of the analytical approach. The agreement between our empirical estimates and literature values further supports the robustness of these methods. For example, the β estimate for the Mexico NM station using method B (0.729 %hPa) closely matches [36], while method A falls within their reported uncertainty (±0.05 %/hPa). Similarly, the results of both empirical methods for the Jungfraujoch NM match closely ($\beta_{EM-A}$ of 0.700 %hPa and $\beta_{EM-B}$ of 0.705 %hPa for the IGY installation) with an analytical function based on a multi-year latitude survey (0.703 %hPa) [24,25], while the analytical values are higher ($\beta_{A}$ of 0.737 %hPa and $\beta_{eff}$ of 0.738 %hPa). Also, [37] reported a β value (0.743 ± 0.017) for Alma-Ata site which was within the range of β estimated in our work from all the methods ($\beta_{EM-A}$: 0.711; $\beta_{EM-B}$: 0.721; $\beta_{eff}$: 0.726).

Beyond the relationships among methods themselves, the PCA also reveals how environmental variables associate with β estimates across the spatial domain. These associations reflect how site characteristics co-vary with β patterns rather than establishing direct causal influences. Altitude shows a positive association with β, projecting similarly along PC1. Sites at higher elevations tend to yield higher β values across all methods. This is physically plausible: reduced atmospheric mass at elevation increases the relative contribution of pressure variations to neutron intensity changes. Atmospheric humidity (AH) exhibits a negative association, projecting in the opposite direction along PC1. Sites with higher humidity tend to have lower β values, aligning with physical expectations: water vapor attenuates cosmic-ray neutrons and may buffer pressure-related intensity fluctuations. The altitude–humidity gradient constitutes the dominant axis of environmental variation (PC1) in our dataset. The observed behavior of altitude-humidity is consistent with cosmic-ray transport theory and previous studies emphasizing altitude–humidity control on β [2,28,38]. Moreover, the PCA results for cut-off rigidity (CR) showed a complex dual relationship with β. CR opposes the β vectors along PC1 (negative correlation) but shares the same direction along PC2 (both project into negative PC2 space). These two variables share a common underlying trend but are distinguished by a specific ‘contrast’ captured along the primary altitude–humidity gradient (PC1). This contrast aligns with theoretical expectations, as sites with higher geomagnetic shielding (CR) tend to show lower β values across all methods. Sites with higher CR receive a harder (higher energy) cosmic-ray spectrum, which may alter the atmospheric cascade development and pressure sensitivity characteristics. While our network is not optimally distributed across climate zones, limiting climate-specific interpretations, this geomagnetic latitude gradient (PC2: 21.1%) operates as a fundamental physical constraint: equatorial sites (13–17 GV cut-off rigidity) show systematically lower β, while polar sites (0–1 GV) exhibit higher β due to their broader low-energy particle spectrum with greater barometric sensitivity.

In general, the shared variances between β and other variables (cut-off rigidity, absolute humidity, and altitude) from the PCA results are consistent with the conceptual framework proposed by [13], which identifies atmospheric depth, closely related to altitude and pressure, as the dominant factor controlling barometric sensitivity, while geomagnetic shielding (cut-off rigidity) exerts a secondary influence. In our dataset, altitude explains most of the variance captured by PC1, whereas cut-off rigidity contributes to the secondary gradient represented by PC2, along with soil moisture, which likely reflects regional co-variation rather than a direct hydrological effect on β. Although PCA generally shows near orthogonality (negligible association) between shared variance from β methods and humidity, both analytical and empirical approaches would benefit from explicit environmental corrections, particularly for humidity. Additionally, the performance of the analytical method is known to vary with altitude: the original parameterizationwas developed using neutron monitor data primarily spanning mid-altitude sites (~2000 m), where attenuation length is best constrained. At higher (>3500 m) or near-sea-level altitudes, data coverage is sparse, and uncertainties increase, particularly where local humidity or soil moisture exerts additional influence. And for a robust empirical β estimation method, explicit humidity correction remains essential. Our study has two key limitations. First, the analysis was based on a relatively large number of CRNS sites concentrated within a specific range of cut-off rigidity, which may restrict the generalizability of the results to other geomagnetic settings, particularly tropical and equatorial regions where high cut-off rigidity (13–17 GV) and distinct atmospheric dynamics may produce different β sensitivities. Expanding CRNS deployment to these underrepresented regions is essential; recent initiatives, such as WASCAL CONCERT, have installed CRNS sensors in West Africa, which, as measurement records accumulate, will provide valuable data to test empirical β coefficient estimation across the full range of geomagnetic and climatic conditions. Second, the PCA and β comparisons were derived from spatial averages over the study period, meaning that temporal variability in β, humidity, and soil moisture was not explicitly captured. This averaging may obscure short-term influences (e.g., storms and seasonal transitions) that could alter barometric sensitivity at individual sites.

## 5. Conclusions

To the knowledge of the authors, this study presents the most comprehensive land-based assessment of barometric coefficients (β) across CRNS and NM networks, analyzing 71 CRNS sites and 47 NM stations to compare analytical and empirical estimation methods and evaluating their sensitivity to key atmospheric and geophysical variables. The barometric effect β ranged from 0.66 to 0.82 %hPa for NM and from 0.63 to 0.80 %hPa for CRNS. Empirical methods showed substantial spatial variability relative to analytical approaches: $\beta_{EM-A}$ exhibited 2.77 times greater variation than $\beta_{A,eff}$, while $\beta_{EM-B}$ showed 4.57 times greater variation. Internal consistency analysis revealed that empirical methods agreed moderately (r=0.67), compared with a correlation of 0.35–0.45 between empirical and analytical methods. The results from PCA decomposition quantified the relationships underlying β’s spatial variability: altitude and humidity together explained 44.0% of variance (PC1), while cut-off rigidity and soil moisture contributed 21.1% (PC2). The analysis demonstrated that altitude-related variations in atmospheric mass are the primary influence on β across sites. Importantly, despite producing different absolute values and variance structures, all three estimation methods ranked sites similarly, as evidenced by their projection into the same PCA quadrant. The practical significance of accurate β estimation was quantified through sensitivity analysis at Petzenkirchen, Austria, where β variations of approximately 10% across the site’s monthly range (0.69–0.77 %hPa^−1^) translated into systematic soil moisture biases of ±0.01–0.02 m^3^/m^3^. The systematic nature of these biases demonstrates that using inappropriate β values, whether from spatial extrapolation or a theoretical method that neglects local and sensor-specific conditions, introduces persistent errors that cannot be corrected without site-specific recalibration ($N_{0}$). The empirical methods’ ability to capture relevant local and sensor-specific conditions makes them particularly valuable for heterogeneous networks, though explicit humidity corrections would further improve performance in humid environments [34]. These findings highlight the need for site-specific β calibration and caution against universal application of theoretical scaling laws in heterogeneous climatic and geomagnetic settings. Expanding datasets to cover a broader range of altitudes, humidities, and geomagnetic environments, alongside integrating temporal dynamics, will be critical to improving pressure correction in CRNS-based soil moisture estimation and enhancing their use in hydrological and climate studies. Finally, further research should focus on dedicated experiments to determine local β values and to analyze possible influencing factors. This should include co-located regular CRNS installations to validate the proposed empirical methods. Possible approaches include latitude surveys with dedicated measurements in different energy ranges; mobile mini NM measurements at various elevations, as performed in Mexico [36]; and CRNS sites where soil moisture and/or snow areas are either stable or continuously monitored by complementary distributed sensor networks. Additionally, a multilevel component analysis approach could address this limitation by simultaneously decomposing variance into between-site (spatial) and within-site (temporal) components. This would allow for the assessment of whether β variability is primarily driven by site characteristics (elevation, cut-off rigidity, and climate zone) or by temporal dynamics (seasonal cycles and atmospheric events) and whether certain sites exhibit greater temporal stability than others.

## Figures and Tables

**Table 1:** Barometric coefficient values (β) for CRNS and NMD data across methods and regions.

Region	Sensor Type	$\beta_{\mathrm{EM}-A}$	$\beta_{\mathrm{EM}-B}$	$\beta_{A,\mathrm{eff}}$	$\beta_{A}$
US	CRNS	0.71 ± 0.020	0.75 ± 0.040	0.73 ± 0.010	0.73 ± 0.010
Europe	CRNS	0.71 ± 0.021	0.72 ± 0.026	0.73 ± 0.005	0.73 ± 0.006
Global	NM	0.72 ± 0.030	0.72 ± 0.024	0.72 ± 0.001	0.73 ± 0.020

**Figure A5:**
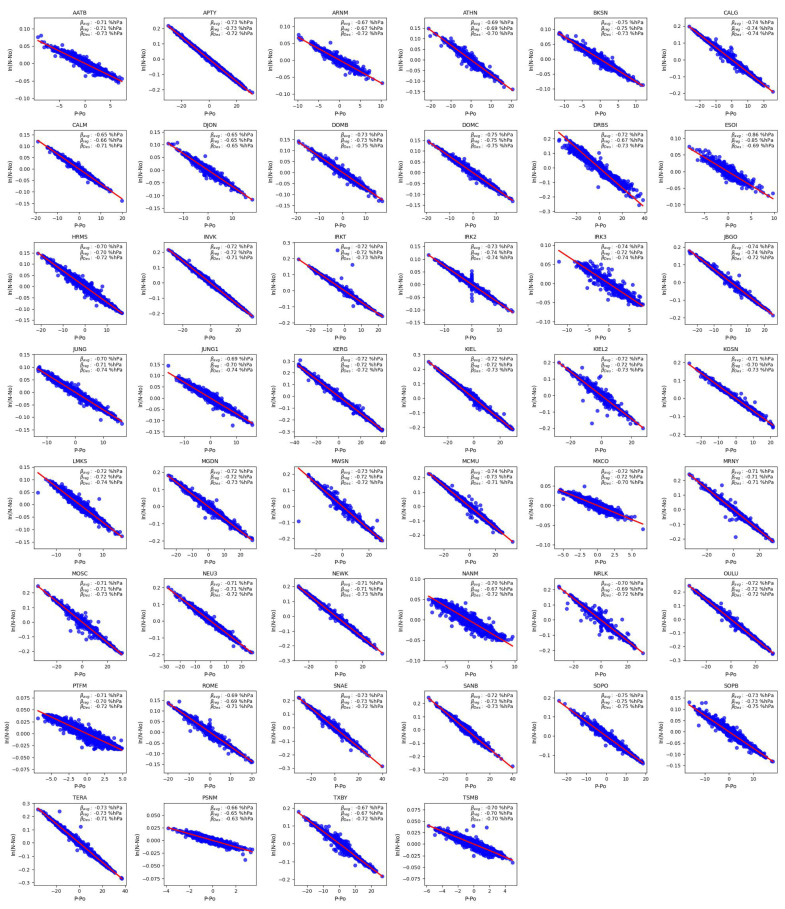
Regression between the relative neutron count rates and atmospheric pressure for all selected 46 NMDB stations. Estimated β coefficients using method A.

## Data Availability

The data used in this study are publicly available. NMDB data can be accessed at https://www.nmdb.eu/nest/ (last accessed on 15 May 2024). Visit the NMDB NEST website, and choose a neutron monitor. Submit your request to view or download the data in ASCII format. For CRNS data for COSMOS-US, the select station is available at https://zenodo.org/records/18412255 [2] (last accessed on 25 January 2026), and the zipped CRNS data for COSMOS Europe are available for download at https://teodoor.icg.kfa-juelich.de/ibg3butt/ibg.butt.download?FileIdentifier=519e0691-7eb3-4351-aff5-c0a0335933ab (last accessed on 24 September 2021) [4].

## Appendix A

**Table A1:** Characteristics of the NMDB stations used in this study and estimated average coefficients ($\beta_{avg}$) using the empirical and analytical methods. Cut-off rigidity (CR) values are from [39].

					Empirical Method		Analytical Method	
Station Name	Lon (°)	Lat (°)	Elev (m)	CR (GV)	$\beta_{\mathrm{EM}-A}$	$\beta_{\mathrm{EM}-B}$	$\beta_{p}$	$\beta_{\mathrm{eff}}$
Alma-Ata B	76.920	43.140	3340	6.610	0.711	0.721	0.723	0.726
Apatity	33.330	67.550	177	0.570	0.729	0.734	0.721	0.721
Aragats	44.170	58.470	3200	7.100	0.681	0.673	0.721	0.723
Athens	23.780	37.980	260	8.530	0.695	0.694	0.697	0.695
Baksan	42.690	43.280	1700	5.600	0.754	0.762	0.736	0.730
CALM	3.160	40.560	708	6.950	0.654	0.665	0.720	0.715
Calgary	−114.130	51.080	1128	1.080	0.743	0.746	0.747	0.736
Daejeon	127.220	36.240	200	11.220	0.647	0.657	0.651	0.654
Dome B	123.300	−75.100	3233	0.010	0.734	0.708	0.768	0.748
Dome C	123.300	−75.100	3233	0.010	0.749	0.736	0.768	0.748
Dourbes	4.600	50.100	225	3.340	0.717	0.732	0.728	0.728
Hermanus	19.230	−34.430	26	4.440	0.704	0.703	0.720	0.723
Inuvik	−133.720	68.350	21	0.160	0.720	0.721	0.710	0.713
Irkutsk	104.030	52.470	435	3.640	0.724	0.724	0.733	0.730
Irkutsk 2	100.550	52.370	2000	3.640	0.735	0.743	0.747	0.740
Irkutsk 3	100.550	51.290	3000	3.640	0.736	0.755	0.747	0.742
Jang Bogo	164.200	−74.600	30	0.300	0.739	0.739	0.721	0.719
Jungfraujoch IGY	7.980	46.550	3570	4.490	0.700	0.705	0.737	0.738
Jungfraujoch NM64	7.980	46.550	3475	4.490	0.695	0.702	0.738	0.738
Kerguelen	70.270	−49.350	33	1.140	0.720	0.724	0.723	0.724
Kiel	10.100	54.300	54	2.360	0.718	0.719	0.725	0.726
Kiel 2	10.100	54.300	54	2.360	0.721	0.722	0.725	0.726
Kingston	147.290	−42.990	65	1.810	0.714	0.709	0.734	0.730
Lomnicky Stit	20.220	49.200	2634	3.980	0.719	0.721	0.745	0.740
Magadan	151.050	60.040	220	2.090	0.721	0.725	0.730	0.729
Mawson	62.880	−67.600	0	0.220	0.728	0.726	0.720	0.718
McMurdo	166.600	−77.950	48	0.010	0.735	0.737	0.716	0.713
Mexico	−99.200	19.330	2274	9.530	0.719	0.729	0.712	0.704
Mirny	93.020	−66.550	30	0.030	0.713	0.714	0.716	0.714
Moscow	37.320	55.470	200	2.430	0.706	0.711	0.729	0.728
Neumayer 3	−8.260	−70.650	17	0.400	0.712	0.720	0.724	0.721
Newark	−75.700	39.700	50	2.020	0.713	0.718	0.723	0.725
Nor-Amberd	44.250	40.370	2000	7.100	0.700	0.703	0.727	0.721
Norilsk	88.050	69.260	0	0.580	0.699	0.699	0.718	0.719
Oulu	25.470	65.050	0	0.780	0.723	0.727	0.718	0.720
Potchefstroom	27.090	−26.700	1351	6.980	0.708	0.708	0.726	0.719
Rome	12.520	41.900	60	6.320	0.686	0.685	0.710	0.712
Sanae64	−2.850	−71.670	856	0.730	0.728	0.737	0.727	0.724
Sanae80	−2.400	−70.310	52	0.860	0.723	0.730	0.737	0.730
South Pole	0.000	−90.000	2820	0.090	0.750	0.757	0.768	0.747
South Pole Bare	0.000	−90.000	2820	0.100	0.729	0.743	0.768	0.748
Terre Adelie	140.020	−66.670	45	0.020	0.726	0.728	0.715	0.713
Thailand	98.490	18.590	2565	16.800	0.658	0.644	0.681	0.634
Tixie Bay	128.540	71.360	0	0.480	0.684	0.689	0.715	0.717
Tsumeb	17.580	−19.200	1240	9.120	0.699	0.698	0.711	0.701
Yakutsk	129.430	62.010	105	1.650	0.713	0.717	0.725	0.726

**Table A2:** Characteristics of the selected EU and US CRNS stations with their respective cut-off rigidity (CR) and average monthly estimated beta coefficients ($\beta_{p}$, $\beta_{eff}$) using the empirical and analytical methods.

					Empirical Method		Analytical Method	
Station Name	Lon (°)	Lat °)	Elev (m)	CR (GV)	$\beta_{\mathrm{EM}-A}$	$\beta_{\mathrm{EM}-B}$	$\beta_{p}$	$\beta_{\mathrm{eff}}$
Aas	59.664	10.762	72	1.210	0.640	0.716	0.726	0.726
Acheleschwaig	47.666	10.994	867	4.150	0.736	0.749	0.733	0.733
Alento2	40.311	15.229	671	6.870	0.629	0.619	0.717	0.717
Boernchen	50.819	13.800	571	3.100	0.731	0.769	0.733	0.733
Calderona1	39.708	−0.457	785	7.360	0.676	0.683	0.714	0.714
Cunnersdorf	51.370	12.557	140	2.940	0.724	0.723	0.729	0.729
Derlo	52.170	23.369	129	2.790	0.720	0.751	0.729	0.729
Elsick	57.039	−2.186	95	1.610	0.701	0.719	0.728	0.728
Esterberg	47.516	11.158	1267	4.250	0.708	0.708	0.735	0.735
Euston	52.383	0.785	18	2.690	0.685	0.717	0.728	0.728
Fendt	47.832	11.060	595	4.080	0.722	0.739	0.731	0.731
Fincham	52.618	0.511	15	2.650	0.693	0.713	0.728	0.728
Fuerstensee	53.319	13.122	66	2.510	0.699	0.701	0.729	0.729
Grosses Bruch	52.030	11.105	80	2.850	0.734	0.735	0.729	0.729
Gludsted	56.071	9.334	86	1.870	0.685	0.699	0.728	0.728
Graswang	47.571	11.033	863	4.230	0.724	0.751	0.733	0.733
Glensaugh	56.914	−2.562	399	1.590	0.707	0.717	0.732	0.732
Harrild	56.022	9.155	66	1.870	0.682	0.695	0.728	0.728
Hordorf	51.997	11.179	82	2.870	0.715	0.725	0.729	0.729
Serrahn	53.339	13.174	96	2.500	0.715	0.50	0.729	0.729
Hohes Holz	52.090	11.226	217	2.810	0.729	0.737	0.730	0.730
Jena	50.951	11.625	140	3.090	0.715	0.725	0.729	0.729
Lullington	50.794	0.189	199	3.030	0.674	0.697	0.729	0.729
Merzenhausen	50.930	6.297	91	3.090	0.707	0.725	0.728	0.728
Petzenkirchen	48.155	15.148	278	4.060	0.713	0.760	0.729	0.729
Rietholzbach	47.381	8.993	755	4.220	0.729	0.748	0.732	0.732
Rollesbroich1	50.622	6.304	515	3.100	0.701	0.696	0.732	0.732
Rollesbroich2	50.624	6.305	506	3.190	0.704	0.705	0.733	0.733
Schoeneseiffen	50.515	6.376	611	3.190	0.707	0.730	0.734	0.734
Gevenich	50.989	6.324	107	3.040	0.699	0.734	0.728	0.728
Ruraue	50.862	6.427	100	3.050	0.705	0.728	0.729	0.729
Wildenrath	51.133	6.169	72	2.980	0.722	0.731	0.729	0.729
Heinsberg	51.041	6.104	58	3.030	0.711	0.714	0.728	0.728
Kall	50.501	6.526	505	3.220	0.703	0.706	0.732	0.732
Aachen	50.799	6.025	232	3.150	0.711	0.724	0.730	0.730
Kleinhau1	50.722	6.372	374	3.090	0.726	0.735	0.732	0.732
Kleinhau2	50.718	6.399	394	3.090	0.702	0.772	0.732	0.732
Saerheim	58.761	5.651	91	1.310	0.686	0.699	0.727	0.727
Schaefertal1	51.657	11.043	425	2.910	0.708	0.739	0.732	0.732
Schaefertal4	51.655	11.049	399	2.910	0.730	0.718	0.732	0.732
Selhausen	50.866	6.447	101	3.100	0.714	0.723	0.729	0.729
Sheepdrove1	51.515	−1.458	197	2.900	0.682	0.685	0.730	0.730
Sheepdrove2	51.528	−1.468	204	2.900	0.707	0.702	0.730	0.730
Sheepdrove3	51.523	−1.486	182	2.900	0.707	0.710	0.730	0.730
Voulund	56.036	9.156	67	1.870	0.665	0.683	0.727	0.727
Wildacker	53.330	13.199	96	2.500	0.747	0.717	0.729	0.729
Weisssee	46.873	10.714	2464	4.420	0.671	0.707	0.738	0.738
Wuestebach1	50.503	6.333	605	3.280	0.721	0.736	0.733	0.733
Wuestebach2	50.505	6.331	607	3.280	0.713	0.738	0.733	0.733
Zuelpich	50.692	6.717	158	3.120	0.718	0.749	0.729	0.729
Bondville	−88.290	40.006	219	2.190	0.682	0.721	0.732	0.730
Daniel Forest	−111.509	41.865	2600	2.320	0.706	0.722	0.757	0.747
GLEES	−106.239	41.364	3190	2.420	0.747	0.719	0.756	0.748
Harvard Forest	−72.172	42.538	350	1.930	0.698	0.695	0.735	0.731
Howland	−68.740	45.204	124	1.520	0.687	0.680	0.728	0.727
Iowa Validation Site	−93.684	41.983	316	1.930	0.738	0.817	0.734	0.731
Marshall Colorado	−105.196	39.950	1756	2.740	0.760	0.831	0.751	0.742
Metolius	−121.557	44.452	1253	2.580	0.717	0.773	0.748	0.739
Mozark	−92.200	38.744	219	2.480	0.686	0.703	0.732	0.730
Neb Field 3	−96.441	41.180	363	2.170	0.692	0.691	0.735	0.732
Park Falls	−90.272	45.946	470	1.250	0.714	0.696	0.736	0.731
Petzenkirchen	15.170	48.141	260	4.170	0.725	0.733	0.731	0.728
Reynolds Creek	−116.723	43.121	1880	2.480	0.742	0.782	0.753	0.734
Rietholzbach	8.993	47.381	755	4.340	0.730	0.791	0.737	0.731
Rosemount	−93.090	44.714	260	1.490	0.694	0.740	0.732	0.729
Saskatoon	−106.617	52.133	506	0.860	0.699	0.745	0.735	0.729
Tower Ruin	−109.718	38.117	1565	3.260	0.722	0.768	0.748	0.739
Trail Valley Forest	−133.496	68.723	125	0.100	0.723	0.751	0.718	0.715
Trail Valley Main Met	−133.496	68.723	125	0.100	0.723	0.751	0.718	0.715
UMBS	−84.714	45.560	220	1.310	0.705	0.840	0.731	0.728
York Irrigated Soybean	−97.459	40.934	494	2.250	0.696	0.738	0.738	0.733
